# Selective decreases in regulatory B-cell (Breg) subsets in active uveitis: IL-10^+^CD19^+^CD24^hi^CD38^hi^Bregs in patients and B10 Bregs in mice

**DOI:** 10.1093/intimm/dxag021

**Published:** 2026-05-07

**Authors:** Yi-Hsing Chen, Xiaozhe Zhang, Malihe Eskandarpour, Rose M Gilbert, Sue Lightman, Virginia L Calder

**Affiliations:** UCL Institute of Ophthalmology, Bath Street, London EC1V 9EL, UK; Department of Ophthalmology, Chang Gung Memorial Hospital, Taoyuan, 33305, Taiwan; College of Medicine, Chang Gung University, Taoyuan 333, Taiwan; UCL Institute of Ophthalmology, Bath Street, London EC1V 9EL, UK; UCL Institute of Ophthalmology, Bath Street, London EC1V 9EL, UK; UCL Institute of Ophthalmology, Bath Street, London EC1V 9EL, UK; UCL Institute of Ophthalmology, Bath Street, London EC1V 9EL, UK; Moorfields Eye Hospital, City Road, London EC1V 2PD, UK; UCL Institute of Ophthalmology, Bath Street, London EC1V 9EL, UK

**Keywords:** B10 cells, experimental autoimmune uveitis, transitional 2 marginal-zone precursor cells

## Abstract

Several regulatory B-cell (Breg) subsets have been implicated in autoimmune diseases. We investigated Breg subsets in non-infectious uveitis (NIU) and in mouse models of experimental autoimmune uveitis (EAU). Blood samples from NIU patients with active disease (*n* = 13), in remission (*n* = 37) and healthy controls (*n* = 10) were immunophenotyped for CD19^+^CD24^hi^CD38^lo^ memory B cells, CD19^+^CD24^hi^CD38^hi^-immature Bregs (iBregs), and CD19^+^CD38^int^CD24^int^-mature Bregs. In NIU, levels of peripheral blood iBregs and serum interleukin-10 (IL-10) were decreased in active disease vs. remission (*P* < .01). In quiescent NIU, iBregs were isolated and, when added at increasing ratios to autologous CD3^+^ T cells, downregulated T-cell proliferation. In an EAU model, at peak disease, CD19^+^CD1d^hi^CD5^+^ B10, CD19^+^CD5^+^ B1, CD19^+^CD21^hi^CD24^hi^CD23^+^ transitional two marginal-zone precursor (T2-MZP) and CD19^+^CD21^hi^CD23^−^ marginal-zone B (MZB) Breg subsets were analysed both phenotypically and functionally. In EAU spleens, all Breg subsets investigated were detectable, but the IL-10–expressing B10-Breg subset was immunosuppressive and inversely correlated with disease severity (*P* = .048, *R*^2^ = 0.47). Blood-derived IL-10–expressing B10 and B1 Bregs were increased in both adjuvant controls and EAU but were significantly reduced in clinically milder EAU. In dissociated retinal cells, only the B10-Breg subset was detectable and increased in EAU. Selective Breg subsets may be immunosuppressive, with iBregs in NIU and IL-10–expressing B10 Bregs in EAU.

## Teaser

A role for Breg subsets in autoimmune uveitis

## Graphical Abstract

**Graphical Abstract:**
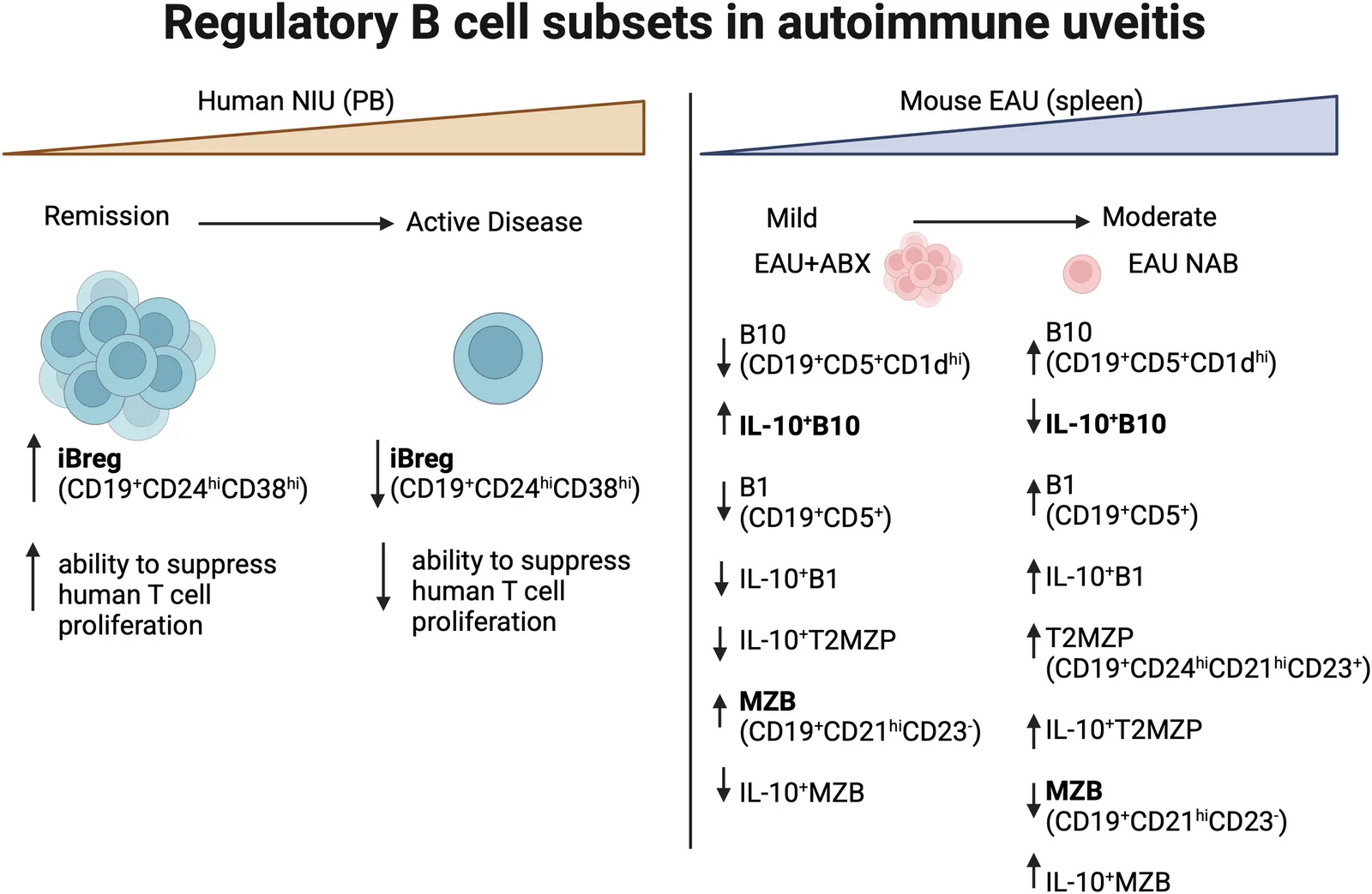

## Introduction

Non-infectious uveitis (NIU) is an autoimmune disease affecting mainly the choroid and retinal tissues of the eye (1). It accounts for 10% of legal blindness in developed countries (2, 3) with a higher prevalence in people of working age (2,4) . Treatment for NIU involves a stepwise approach with corticosteroids, specific T-cell inhibition by immunosuppressants, and biologics targeting specific immune pathways (5–8).

Experimental autoimmune uveitis (EAU), a model recapitulating many features in NIU, is driven by autoreactive Th1 and Th17 cells (9–12). Regulatory T cells (Tregs) suppress effector CD4^+^ T cells (Teff cells) through interleukin-10 (IL-10) and transforming growth factor β (TGF-β) production (13–15) and studies have demonstrated a downregulatory role for Tregs in NIU (16, 17). In a previous study from our group comparing Tregs from a cohort of 37 quiescent and 13 active uveitis patients, it was found that T cell immunoreceptor with immunoglobulin and ITIM domains (TIGIT)-expressing Tregs correlated with disease activity (18). In EAU, only activated Th1 or Th17 cells can adoptively transfer disease into naive mice. B cells are not uveitogenic, and their contribution to disease remains unclear.

B cells display several immune functions: immunoglobulin and cytokine production; antigen presentation; CD4^+^ T-cell regulation. Understanding the roles of regulatory B cells (Bregs) is challenging since Bregs make up <2% of leucocytes in human blood and intraocular levels are even lower (19). However, Bregs have been detected in retinal tissues in both NIU and EAU (19, 20) and are thought to mediate their suppressive function via IL-35 and/or IL-10 production (21, 22).

In mice, various Breg subsets have been defined, including transitional two marginal-zone precursor (T2-MZP) cells, CD5^+^CD1d^hi^-B10 cells, marginal-zone B (MZB) cells, Tim-1^+^ B cells, CD138^+^ plasma cells, and plasmablasts (22, 23). In humans, CD19^+^CD24^hi^CD38^hi^immature Bregs (iBregs), CD19^+^CD38^int^CD24^int^primary mature Bregs, and CD19^+^CD24^hi^CD38^−^primary memory B cells have been identified (24–26). Previously, the majority of iBregs were phenotyped as IgM^hi^IgD^hi^CD5 ^+^ CD10 ^+^ CD20 ^+^ CD27^−^CD1d^hi^, whereas primary mature Bregs were IgM^int^IgD^+^CD5^−^CD10-CD20 ^+^CD27^−^CD1d^+^. Primary memory B cells were also defined as CD5^−^CD10^−^CD27^+^CD1d^+^ and existed as IgM^+^IgD^+^ and IgM^−^IgD^−^ subtypes (25). Dysfunctional iBregs were reported as having impaired IL-10 production and reduced ability to suppress Teff cells and Treg differentiation in systemic lupus erythematosus (SLE) and rheumatoid arthritis (RA) patients (25, 26). It has been suggested that different inflammatory environments promote distinct Breg subsets.

To determine the level and function of different Breg subsets, we analysed blood samples from the abovementioned cohort of patients with active or quiescent NIU, and healthy controls (18). Prior studies demonstrated that short-term depletion of commensal microbiota in mice significantly attenuated EAU (27, 28). We therefore addressed the effect of commensal microbiota in promoting Breg subsets by comparing EAU with EAU mice pre-treated with a short-term course of antibiotics (ABX) to deplete their microbiota.

## Methods

### Patient recruitment and sample collection

Patients aged between 18 and 59 years were recruited prospectively according to their Standardization of Uveitis Nomenclature–classified disease activity (29). The study was conducted in accordance with recommendations of the UK National Research Ethics Service-London Harrow Committee and the Moorfields Eye Hospital National Health Service Foundation Trust, Department of Research and Development (13/LO/1653; 16039). Informed consent in accordance with the Declaration of Helsinki was received from all subjects. ‘Active’ disease was defined as newly diagnosed or onset NIU. ‘Remission’ was designated for inactive NIU at recruitment for >6 months. The clinical data of this cohort were published (18).

Heparinized peripheral blood (PB; 30 ml) was obtained at recruitment and PB-derived mononuclear cells (PBMCs)s isolated by Ficoll density centrifugation before culturing with anti-CD3/CD28 (Dynabeads) for 24 h or with a stimulation cocktail [phorbol 12-myristate 13-acetate (PMA) and ionomycin; 1:500] for 6 h as a positive control, and Brefeldin A (BFA) added for the last 4 h for detecting intracellular cytokine expression (see below).

### EAU induction

Two models of EAU were used in this study. The B10RIII mice developed an acute retinal monophasic disease, which peaked at 10–14 days (d) post-immunization (pi), and then declined by 21d pi. The C57BL/6 EAU model displayed a clinically milder form of retinal inflammatory disease, peaking at 17–21d and then declining to a low level of retinal inflammation lasting several weeks. No spontaneous recurrences were observed in either model. Female B10.RIII mice (in-house colony) and C57BL/6 mice (Charles River Laboratories), aged 6–8 weeks, were used (*n* = 5–8 per experimental group). EAU was induced by immunizing with subcutaneous interphotoreceptor retinoid-binding protein (IRBP) peptides (400 μg; IRBP_161–180_ for B10.RIII mice (30); IRBP_1–20_ for C57BL/6 mice (31)) in complete Freund’s adjuvant (CFA; Sigma, UK) supplemented with 1.5 mg/ml *Mycobacterium tuberculosis* H37Ra (Difco Microbiology, USA). All mice subsequently received 0.4 μg *Bordetella pertussis* toxin (Sigma-Aldrich, UK). Untreated EAU mice [without antibiotics (EAU + NAB)] were compared with mice given oral ABX [ampicillin (1 g/ml), vancomycin (0.5 g/ml), neomycin sulphate (1 g/ml), and metronidazole (1 g/ml)] 7d prior to EAU induction (EAU + ABX) (27). Experiments included non-immunized mice (NI group) and mice immunized with CFA alone (MTB group). Mice were maintained for 21d post-EAU induction and tissues were harvested for analysis.

Studies were conducted according to the UK Home Office Regulations on the Care, Welfare and Treatment of Laboratory Animals and in compliance with the Association for Research in Vision and Ophthalmology Statement on the Use of Animals in Ophthalmic and Visual Research.

### Lymphocyte isolation

PBMCs were isolated from mouse PB by density centrifugation (Lymphocyte-M, Tebu-Bio). Splenocytes were filtered and washed with red blood cell (RBC) lysis buffer. Retinal cells were isolated (32), cultured in 24-well plates, and stimulated with the PMA and ionomycin cocktail for 5 h, adding BFA for the last hour before harvesting for immunophenotyping. Unstimulated cells were used as controls.

### Flow cytometry

For immunophenotyping, data were acquired using BD FACSDiva (v6.1.3). Controls included compensation controls (OneComp/UltraComp beads) and FMO. Cells were incubated with relevant surface antibodies and a Live/Dead fixable dye (Molecular Probes, Life Technology, UK) for 30 min at 4°C. Cells were then permeabilized using fixation/perm buffer (eBioscience, La Jolla, CA, USA) for 10 min before adding directly conjugated intracellular mAbs for a further 30 min and a final wash. A total of 100 000 events were recorded per sample through a live, single cell gate.

Subsets of human Bregs were identified by staining with directly conjugated mAbs recognizing CD3 (Violet Blue, Miltenyi, UK), CD19 (BV605), CD24 (PE-Cy7), and CD38 (PE-Dazzle594, all BioLegend) to identify the Breg subsets: CD19^+^CD24^hi^CD38^lo^ (primary memory B cells), CD19^+^CD24^int^CD38^int^ (primary mature B cells), CD19^+^CD24^hi^CD38^hi^ (iBregs), and CD19^+^CD24^low^CD38^hi^ (plasmablasts; all BD Biosciences, UK; Supplementary Figure 1). For detecting intracellular IL-10, a directly conjugated anti-human IL-10 mAb (PE-CF594; B-D) was used.

For mouse Bregs, directly conjugated mAbs recognizing CD19 (BUV395), CD5 (PerCP/Cy5.5), CD1d (AF647), CD21 (PE/Cy7), CD23 (PE), CD24 (BV605), and IL-10 (BV421; all BioLegend) were used to characterize levels of B10-CD19^+^CD1d^hi^CD5^+^, B1-CD19^+^CD5^+^ (Supplementary Figure 2), T2-MZP-CD19 ^+^ CD21^hi^CD24^hi^CD23^+^, and MZB-CD19^+^CD21^hi^CD23. Intracellular IL-10 expression was detected by anti-mouse IL-10 mAb (BV421; B-D). FlowJo (v10.2) was used for flow cytometric analysis.

### Breg suppression assay

For evaluating human iBreg function, proliferation assays of effector cells (Teff cells) were performed with autologous iBregs after isolating Bregs by direct cell sorting using CD3 (BV785), CD19 (AF488), CD38 (APC), CD24 (PE) surface Abs (Supplementary Figure 3).

For assaying mouse B10-Breg function, *in vivo*–primed effector cells (Teff cells: CD3^+^CD4^+^CD25^−^CD127^lo^) were isolated from C57BL/6J EAU lymph nodes and sorted by CD3 (AF488), CD4 (PE/Cy7), CD25 (BV605), CD45RB (AF647), and CD127 (PE-CF594) using BD Influx (BD Biosciences, USA). The surface markers CD19 (PerCP/Cy5.5), CD1d (PE), CD5 (BV421), and DRAQ7 (NIR) were used to isolate splenic B10 Bregs by direct sorting as above (Supplementary Figure 4).

For the assay, Teff cells were labelled with carboxyfluorescein succinimidyl ester (CFSE) (33) before plating in triplicate at 1 × 10^5^ per well at ratios of 1:0.25, 1:0.125, 1:0.0625, in 96-well, round-bottom plates in complete culture medium, stimulating with plate-bound CD3 antibody in medium containing CD28 antibody. Wells containing Teff cells alone were used as controls. On Day 5, cells were harvested for flow cytometric analysis using Violet Proliferation Dye signal (LSR Fortessa X-20, BD Bioscience, UK). ModFit software (Verity Software House, USA) was used to calculate the percentages of proliferating cells.

### Serum cytokines

Whole blood samples were centrifuged and serum isolated and stored frozen (−70°C) in aliquots. Freshly defrosted serum samples were assayed for IL-10 and IL-22 by multiplex bead arrays (R&D Systems) using a MAGPIX Luminex system (Luminex, TX, USA), according to the manufacturers’ instructions. Data were analysed with xPONENT software (Luminex). Standard curves were plotted, and minimum levels of detection were IL-10 (7.30 pg/ml) and IL-22 (6.10 pg/ml).

### Statistical analysis

Results were presented as the mean ± standard error of the mean (SEM) for at least 5 data points or mean ± SD for 3 data points. Differences between more than three groups were determined by the Kruskal–Wallis tests with *post hoc* Bonferroni correction for multiple comparisons. For mouse experiments, Student’s *t*-tests were used for comparing means. Bivariate correlations between immunological variables were calculated using Spearman’s test. The coefficient of determination, *R*^2^, was used to represent the proportion of the variance in the dependent variable that is predictable from the independent variable. Relationships between selected variables, which had clinically relevant associations, were modelled using linear regression. Statistical analysis of final datasets was performed with SPSS version 24 (IBM Software) and GraphPad Prism version 10.4.1. All significance tests were two-tailed. *P* values of <.05 were considered significant.

## Results

### iBregs correlated with NIU disease remission

Fifty patients with idiopathic, non-anterior NIU (37 remitted and 13 active) and 10 age-matched healthy (control) subjects without systemic comorbidities were recruited from Moorfields Eye Hospital, London, UK (2014–16). Of those in remission, 22 (59%) had prior corticosteroid-only therapy, the other 15 (41%) received additional immunosuppressants. The anatomical diagnoses of intermediate [6 (46%) in the active group and 16 (43%) in the remitted group], posterior uveitis [1 (8%) in the active group and 6 (16%) in the remitted group], or panuveitis [6 (46%) in the active group and 15 (41%) in the remitted group] were evenly distributed across the active and remission groups. Among the active NIU patients, six (46%) received immunosuppressants at baseline and four underwent additional immunophenotyping during follow-up.

**Figure 1.**
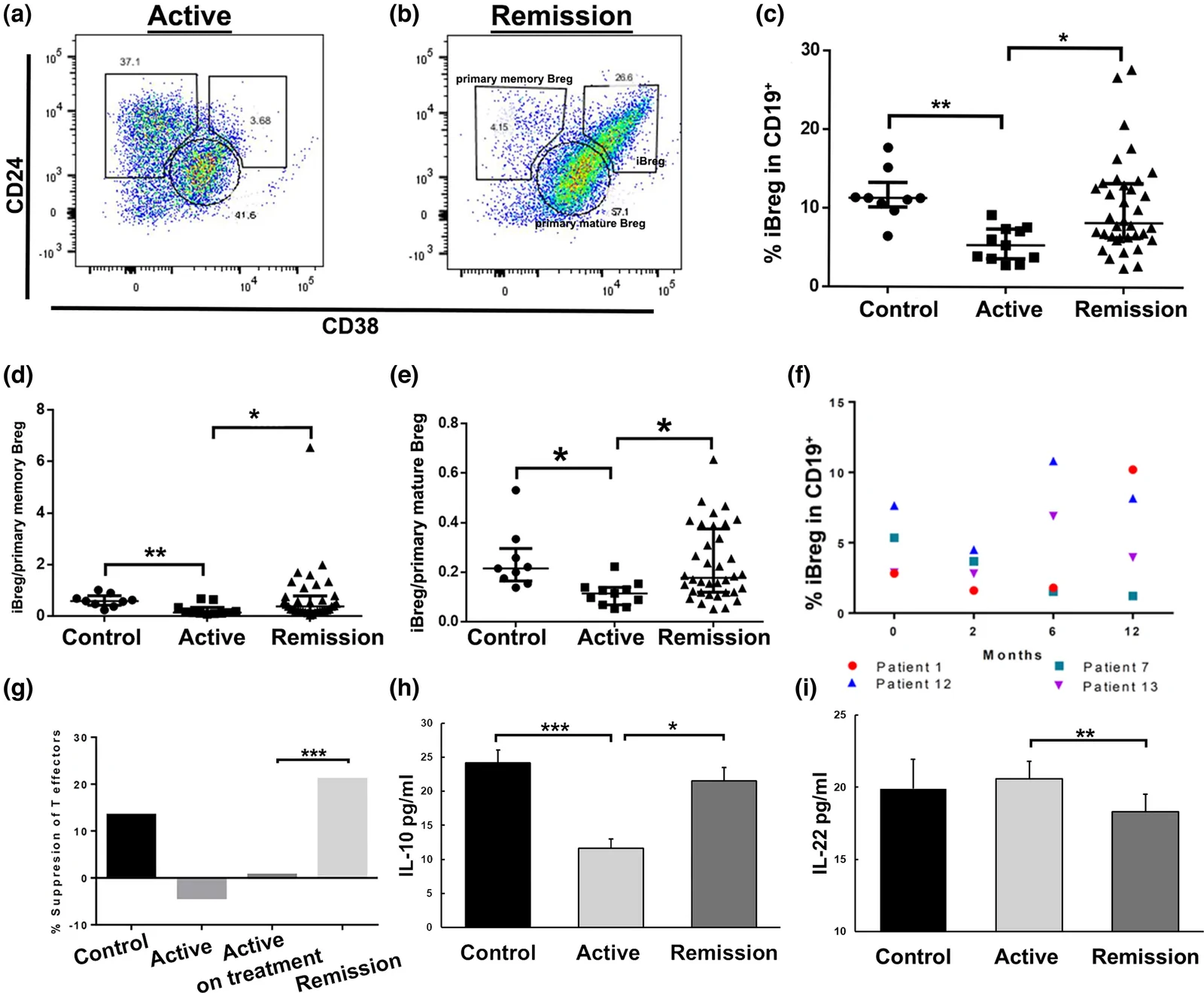
Human PB-derived Breg subsets in NIU patients. Examples of CD24 and CD38 expression in CD19^+^ cells in an active patient (a) and in a remitted patient (b). (c) CD19^+^CD24^hi^CD38^hi^ immature iBreg expression in control, active, and remission uveitis patients’ cohorts. Ratio of iBreg/CD19^+^CD24^hi^CD38^lo^-primary memory Bregs (d) and iBreg/CD19^+^CD24^int^CD38^int^-primary mature Bregs (e) in control, active and remitted uveitis patient cohorts. (f) The changes in levels of CD19^+^CD24^hi^CD38^hi^ iBreg cells during 12 months in Patients #1, #7, #12, and #13 with initial active uveitis. Patients #7 and #13 had poor treatment response to immunosuppressants, whilst Patients #1 and #12 illustrated good clinical response to treatment at Month 12. (g) iBreg % suppression of Teff cells’ proliferation from control (*n* = 5), active (*n* = 7), active on treatment (*n* = 6), and remission groups (*n* = 10). (h, i) Serum levels from control (*n* = 5), active (*n* = 7), and remission (*n* = 10) groups showing mean levels of IL-10 (h) and IL-22 (i). Data were presented as mean ± SEM. *P < .05; **P < .01; ***P < .001 using a two-tailed, unpaired Student’s *t*-test.

The Breg subsets were gated as shown (Fig. 1a and b). Levels of iBregs from active disease were significantly lower than controls (*P* < .01) and remission (*P* < .05; Fig. 1c). Levels of primary memory Bregs and primary mature Bregs were similar between the three groups (data not shown). The ratio of iBregs to primary memory Bregs in the active group was significantly lower compared with controls and patients in remission (Fig. 1d). The ratio of iBregs to primary mature Bregs was also lower in the active group compared with control and remission groups (Fig. 1e). Whilst we had a similar prevalence of intermediate, posterior, and panuveitis across the two patient groups, we did not observe any correlation between clinical diagnoses and levels of Bregs. Similarly, we did not observe any correlation between treatment and Breg levels.

Four patients from active NIU at baseline (#1, #7, #12 and #13) participated in follow-up examinations at 2, 6, and 12 months. At 12 months, #1 and #12 achieved good clinical remission whilst #7 and #13 remained active. Immunophenotyping suggested that iBregs increased as the disease resolved in #1 and #12 but remained low in active patients #7 and #13 (Fig. 1f). A similar trend was noted in a patient’s blood, investigating IL-10 expression in iBregs, where levels increased to those of a control following treatment at 6 months (Supplementary Figure 1h).

### Human iBregs suppressed autologous Teff cells in vitro

To investigate their immunosuppressive function, iBregs were isolated from NIU blood samples to compare with those from controls (Supplementary Figure 3). The results showed that iBregs from control blood samples were able to inhibit Teff cells’ proliferation *in vitro* by ∼13.5% (Fig. 1g). However, iBregs from active NIU blood samples, naive to treatment, and iBregs from active NIU on treatment failed to suppress Teff-cell proliferation (Fig. 1g) when compared with Bregs from active NIU without treatment. The iBregs from NIU in remission demonstrated the highest *in vitro* functional suppression of Teff cells when compared with the active NIU on treatment group (*P* < .001). Serum from the same patient groups showed a significant decrease in IL-10 levels in active NIU when compared with control and remission groups, suggesting a potential role for this anti-inflammatory cytokine (Fig. 1h). This contrasted with IL-22 from the same serum samples, where the remission group had significantly lower levels than controls and active NIU (Fig. 1i). Altogether the data suggest that NIU in remission was associated with increased levels of iBregs and IL-10 serum levels in comparison with active NIU.

### Differential effects on splenic Breg subsets from gut dysbiosis in EAU

**Figure 2.**
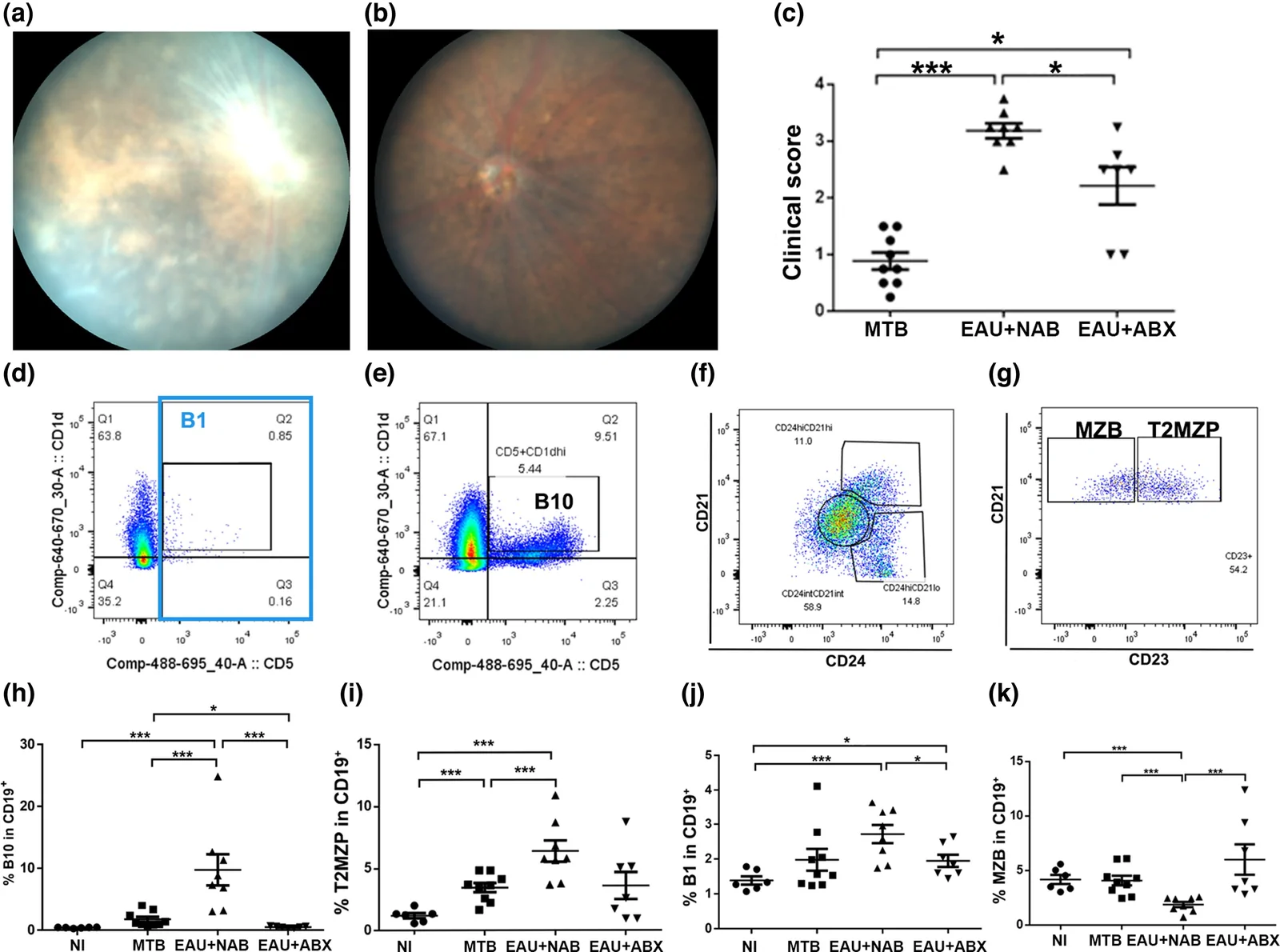
Phenotypic splenic regulatory B-cell (Breg) subsets in EAU. Examples of Day 21 colour fundus photo of C57/BL6 EAU mice (NAB, (a)) and receiving antibiotics (ABX, (b)). (c) Clinical scores of peak stage EAU mice were downregulated following ABX treatment compared with NAB controls. (d, e) Example of gating strategy for CD1d^+^ and CD5^+^ in CD19^+^-gated, live intraocular single cells in the EAU + ABX group (d) and in the EAU + NAB group (e). (f) Pattern of CD21 and CD24 distribution in CD19-gated cells. (g) Distribution of CD21 and CD23 in the CD19^+^CD21^hi^CD24^hi^ gated region. (h–k) Distribution of CD1d^hi^CD5^+^CD19^+^ (B10) cells (h); CD19^+^CD21^hi^CD24^hi^CD23^+^ (T2-MZP) cells (i); CD19^+^CD5^+^ (B1) cells (j); CD19^+^CD21^hi^CD23^−^ MZB Breg cells (k) in EAU + NAB, EAU + ABX groups compared with MTB (mice immunized with MTB and pertussis), and non-immunized (NI) mice. Representative data of three independent experiments are shown. Data were presented as mean ± SEM. *P < .05; ***P < .001 using a two-tailed, unpaired Student’s *t*-test.

To investigate whether there is an association between Breg levels and the intestinal microbiome, C57BL/6 mice were treated with oral ABX for 7d prior to inducing EAU. Our data showed that at peak EAU, disease scores were significantly downregulated following ABX treatment (Fig. 2a–c). Interestingly, in the more severe B10.RIII model of EAU, ABX pre-treatment resulted in a significant decrease in the total bacterial counts relative to ABX-treated controls which correlated with decreases in certain pathogen loads in the stool samples, including *Lactobacilli* spp., *Streptococcus intermedius*, and *Staphylococcus xylosus* (Supplementary Figure 5), confirming previous studies demonstrating that gut microbiome contributes to EAU severity (34, 35).

We then analysed splenic Bregs at peak EAU in C57BL/6 mice (Fig. 2d–g). We detected four Breg subsets: B10, T2-MZP, B1 and MZB cells. The results highlighted that B10, T2-MZP, and B1 Breg levels were significantly increased during EAU + NAB. EAU + ABX treatment resulted in decreased levels of the B10 and B1 Breg subsets when compared with MTB controls (Fig. 2h–j). In contrast, MZB Bregs were significantly increased following ABX treatment (EAU + ABX; Fig. 2k), suggesting that specific gut microbes could differentially regulate this MZB Breg subset.

### Increased IL-10–expressing splenic B10-Breg levels were associated with decreased severity of EAU and depletion of gut microbiome

**Figure 3.**
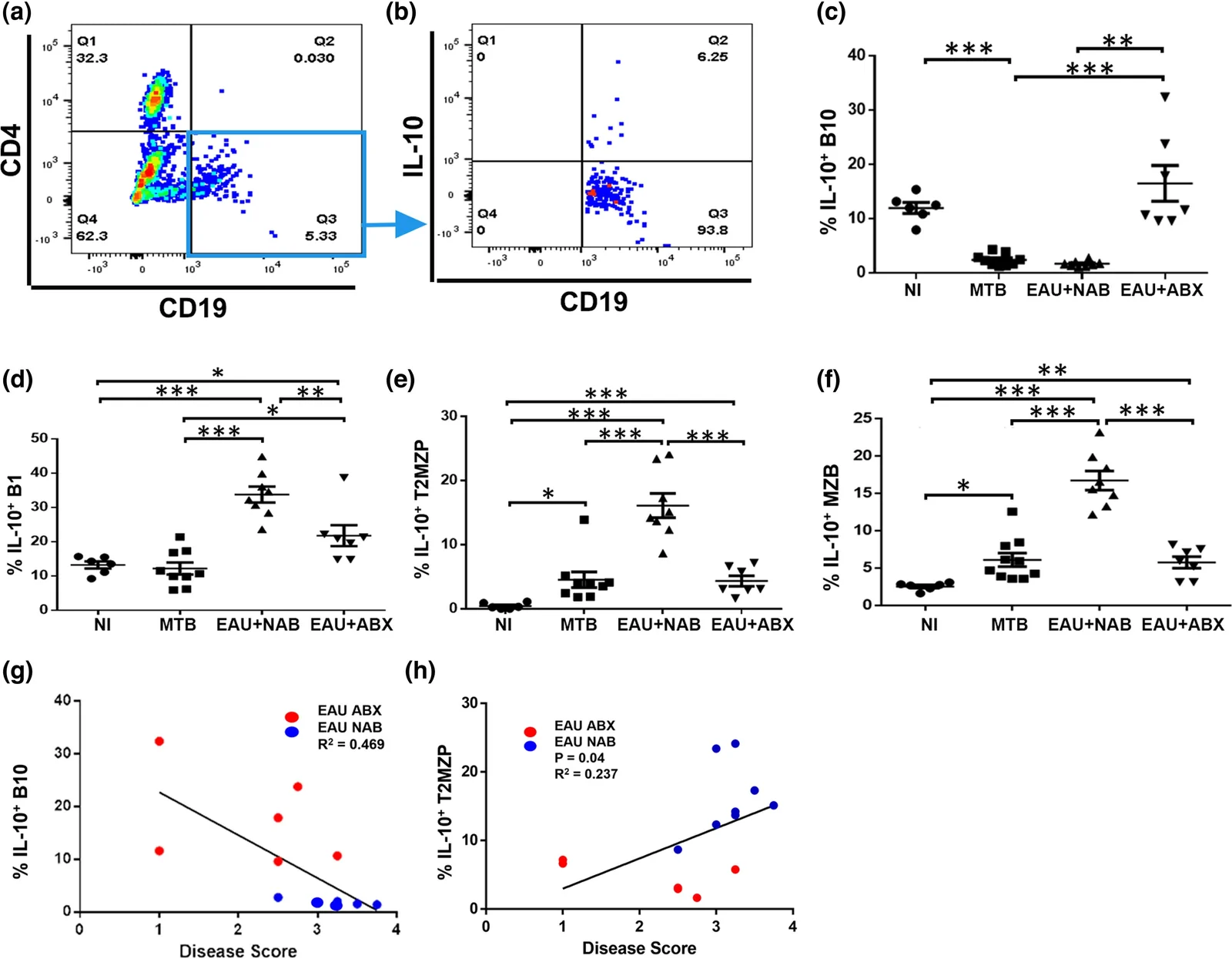
Detecting splenic Breg cells in C57BL/6 EAU mice. (a) Flow cytometry scatter plot demonstrating the distribution of CD4^+^ T and CD19^+^ B cells in splenic single cells from EAU mice treated with antibiotics (ABX). (b) Flow cytometry scatter plot demonstrating the IL-10–producing CD19^+^ B cells in the splenic single cells from an EAU + ABX group mouse. (c–f) Distribution of IL-10–producing B10 (c), B1 (d), T2-MZP (e), MZB (f) cells in non-immunized (NI), MTB (mice immunized with MTB and pertussis), EAU mice treated with no antibiotics (NAB) and antibiotics (ABX). (g) Negative correlation of IL-10–producing B10 cells and disease score at Day 21 of EAU induction (*P* = .0048, *R*^2^ = 0.469). (h) Positive correlation of IL-10–producing T2-MZP Breg cells and EAU severity score at Day 21 of EAU induction (*P* = .04, *R*^2^ = 0.237). Data were presented as mean ± SEM. *P < .05, **P < .01, ***P < .001 using a two-tailed, unpaired Student’s *t*-test.

Having demonstrated a downregulation of EAU severity with ABX pre-treatment, it was unclear which of the four Breg subsets might be responsible for this effect since all were detectable at varying levels. Within mouse CD19^+^ B-cell subsets (Fig. 3a), we next investigated intracellular IL-10 expression as a marker of regulatory function and found that CD19^+^ B cells expressed low levels of IL-10 (≅6.25%; Fig. 3b). However, by analysing IL-10 expression in Breg subsets, we found increased levels of IL-10–expressing B10 Bregs were associated with ABX treatment in comparison with EAU + NAB and MTB controls (Fig. 3c). In contrast, levels of IL-10–expressing T2-MZP, B1, and MZB cells were all decreased following ABX treatment compared with NAB controls (Fig. 3d–f). The disease severity scores were inversely correlated with splenic IL-10 expression by B10 Bregs (*P* < .0048; Fig. 3g), suggesting that lower EAU disease scores could be attributed to the downregulatory effect of IL-10–expressing B10 Bregs. In contrast, the percentages of IL-10–expressing T2-MZP Bregs were positively correlated with EAU disease severity (*P* = .04; Fig. 3h), suggesting that this Breg subset was unable to suppress disease. IL-10–producing B1 and MZB Breg levels did not significantly correlate with EAU disease activity (data not shown).

### Breg subsets in PB and retinal cells in EAU

**Figure 4.**
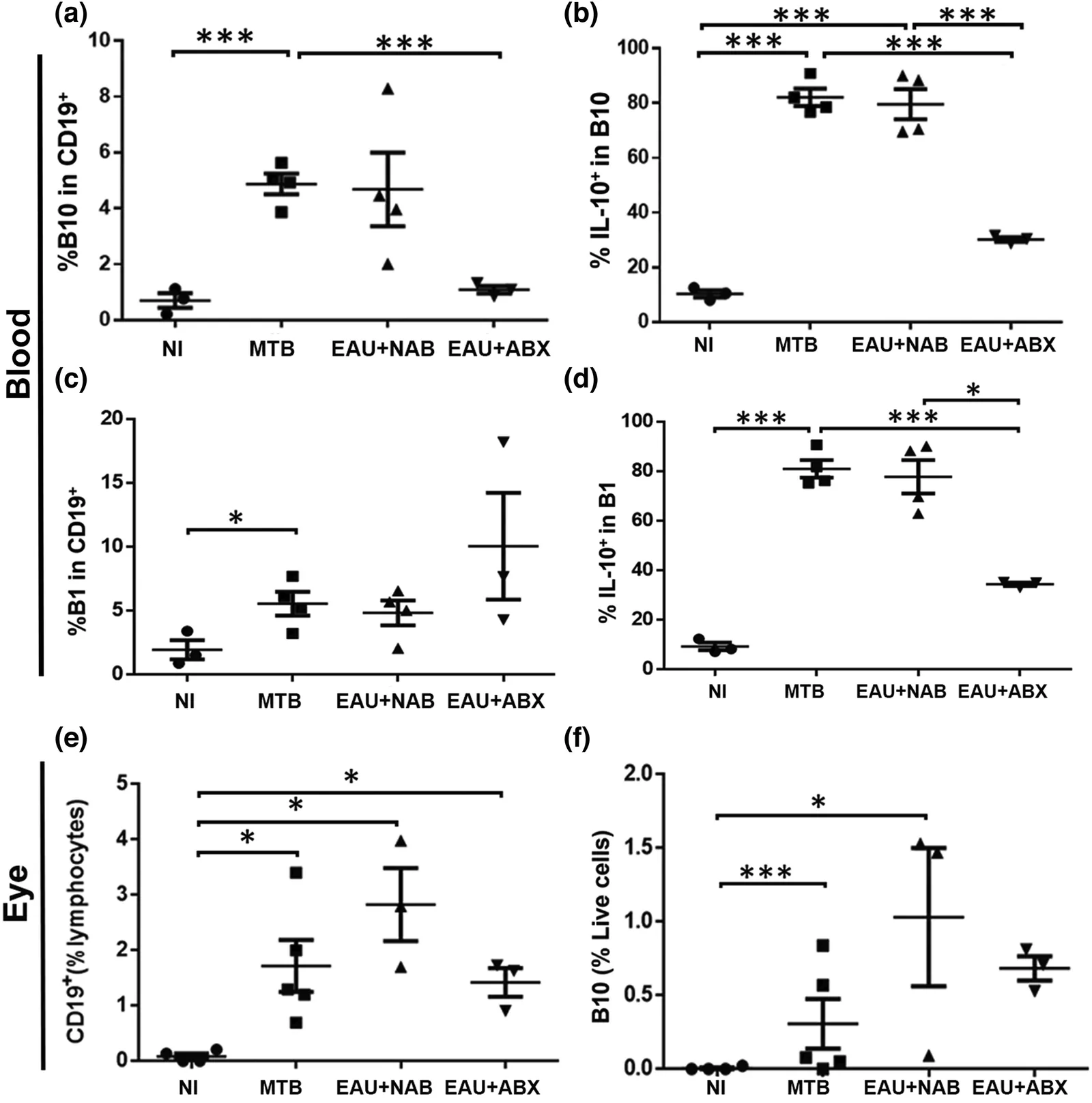
Detecting Breg subsets in PB and retinae in C57BL/6 EAU mice. (a–c) Phenotypic B10 (a) and B1 (c) Breg cell subsets in blood samples from each EAU group compared with controls. (b–d) IL-10 expression in B10 (b) and B1 (d) cells within the blood in each group. (e) Total intraocular B cells (CD19^+^) from each EAU group compared with controls. (f) Phenotypic B10 cells within the intraocular live cells from each EAU group compared with controls. Representative data of three independent experiments are shown. Data were presented as mean ± SEM. *P < .05; ***P < .001 using a two-tailed, unpaired Student’s *t*-test.

Breg subsets were also assessed in the PB at peak stage of EAU. Both B10 (Fig. 4a) and B1 (Fig. 4c), Bregs were significantly increased in the MTB group relative to non-immunized controls. Whilst ABX treatment decreased PB levels of B10 cells, the B1 cell levels were not significantly altered (Fig. 4a and c). We detected increases in IL-10–expressing B10 and B1 Bregs in both the MTB and EAU + NAB groups when compared with the non-immunized (naive; NI) group (Fig. 4b and d). The levels of IL-10–expressing B10 and B1 cells were decreased in EAU + ABX relative to MTB and EAU + NAB groups (Fig. 4b and d). T2-MZPs and MZB cells were undetectable in the PB at peak EAU (data not shown).

In dissociated retinal cells, increases in CD19^+^ B cells in the MTB, EAU + NAB and EAU + ABX groups were detected relative to NI controls, albeit at relatively low levels (Fig. 4e). Upon further analysis of Breg subsets, the levels of B10 cells were increased in both NAB and MTB groups when compared with NI controls (Fig. 4f). Retinal B1 cells were unchanged in all groups (data not shown). Intracellular IL-10–expressing Bregs were undetectable due to low levels in the eye.

### Mouse B10 Bregs suppressed autologous Teff cells *in vitro*

**Figure 5.**
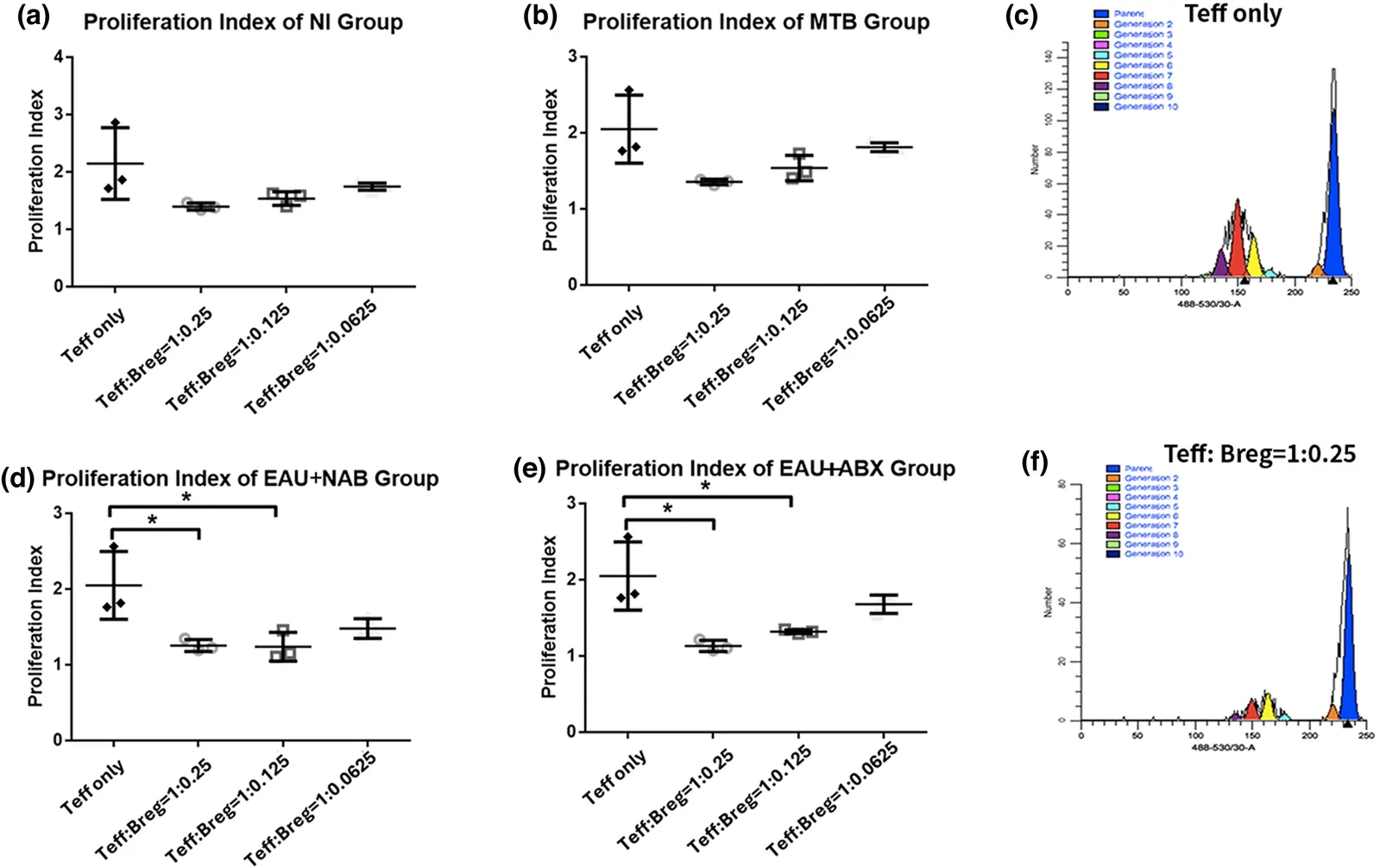
Functional suppression by regulatory B cells (Bregs) isolated from spleens and co-cultured with Teff cells from C57BL/6 peak-EAU mice. (a, b) The results showed that proliferating Teff cells were not suppressed by Bregs isolated from NI (a) and MTB (b) groups at the ratio of 1:0.25, 1:0.125, and 1:0.0625. (c) Representative proliferation from Teff cells only. (d, e) The results showed that proliferating Teff cells were suppressed by Bregs isolated from EAU + NAB (d) and EAU + ABX (e) groups at the ratio of 1:0.25 and 1:0.125. (f) A representative result from EAU + ABX mice with Teff: Breg = 1:0.25 ratio. Data were presented as mean ± SD. *P < .05 using a two-tailed, unpaired Student’s *t*-test.

To investigate whether mouse B10 Bregs were functionally immunosuppressive *in vitro*, splenic B10 cells were isolated from each group, and Teff cells prepared from EAU lymph nodes. In the absence of B10 Bregs, Teff cells proliferated in response to anti-CD3/28 stimulation (Fig. 5c). After 5d’ co-culture of Teff cells with B10-Bregs, there was a trend for the B10-Bregs from NI and MTB groups to downregulate Teff-cell proliferation, but this did not reach significance (Fig. 5a and b). In contrast, proliferation of Teff cells was significantly decreased in the presence of the B10 Bregs from both EAU groups: EAU + NAB (Fig. 5c and d) and EAU + ABX (Fig. 5e and f). Comparing the CFSE peaks obtained (Fig. 5c and f), levels of proliferating Teff cells in the presence of B10 Bregs from the EAU + ABX group (Fig. 5f) were decreased by 75% in the absence of B10 Bregs (Fig. 5c), supporting their anti-proliferative effect.

## Discussion

Our study investigated Breg subsets in PB from active and quiescent NIU patients who did not have any systemic comorbidities. In NIU, decreases in percentages of iBregc were associated with active disease, suggesting that immunoregulatory function of iBregs was related to quantity. In EAU, phenotypically distinct B10 and B1 Breg subsets were detected in spleen, retina, and PB. The results indicated that percentages of splenic IL-10–expressing B10 cells were inversely associated with EAU disease scores, suggesting that this subset may be downregulating EAU. On the other hand, T2-MZP and MZB Breg subsets were only detectable within the spleen and were associated with high EAU disease scores, suggesting they were ineffective in immunoregulating EAU, perhaps because of insufficient numbers or their localization to the spleen. A summary of the relative Breg changes with regards to the disease severity in human NIU regardless of the clinical subtype of uveitis and in mouse EAU suggests that IL-10–expressing human iBregs and mouse B10-Bregs are inversely associated with disease severity.

The role of Bregs in autoimmune disease remission is well documented in SLE (36), experimental autoimmune encephalitis (EAE) (37), and in models of colitis (38) and arthritis (39, 40). In man, the level of B10 Bregs was reported to inversely correlate with disease activity scores and the level of C-reactive protein in patients with RA, suggesting its immunoregulatory role (41). B10 Bregs have also been reported to be immunosuppressive in a murine lupus model and in EAE (36, 42). In EAE, only antigen-specific B10 exerted an anti-inflammatory effect, whilst other subsets of Bregs promoted disease progression (37). In an *in vivo* arthritis model, however, the suppressive effect of T2-MZP Bregs was paralleled by an inhibition of antigen-specific T-cell activation and impaired Th1-cell function (43). Furthermore, mesenteric T2-MZPs only partially suppressed Teff cells, suggesting a differential immunoregulatory capacity of Breg subsets (44). Our data support the hypothesis that only IL-10–producing B10 cells are immunoregulatory for EAU, whilst T2-MZP and MZB Bregs may be proinflammatory.

In RA patients, iBregs were found to perform two functions: to limit the production of proinflammatory cytokines by firstly preventing CD4^+^ T-cell commitment into Th1 and Th17 subsets and, secondly, by converting Teff cells into Tregs (26). It has been reported that CD38 promotes FoxP3 expression and the immunosuppressive function of Tregs in a murine model of SLE and in patients with multiple myeloma (45–47). It is possible that iBregs participate in Treg activation through CD38 expression. In SLE blood samples, iBregs were found to suppress Th1-cell function via secretion of IL-10 but not TGF-β (25). It has been suggested that iBregs (41) or the CD24^hi^CD27^+^ B-cell subset are the precursors of B10 cells (24). Our findings of increases in levels of iBregs and serum IL-10 during remission support previous work demonstrating numbers and regulatory function of PB iBregs were increased during disease remission.

It has been shown that IL-10 production is essential for Breg immunoregulatory function, as B cells isolated from IL-10 knockout mice failed to mediate a protective function (24, 39). In healthy PB, iBregs downregulated Teff-cell proliferation, secretion of interferon-γ (IFNγ) and tumour necrosis factor-α, partially via production of IL-10 (26). Our results also demonstrated that IL-10 is involved in Breg immunomodulation, although our data do not rule out a role for TGF-β.

STAT3 deletion in mouse B cells resulted in exacerbated EAU and EAE, enhanced expression of costimulatory molecules on B cells, marked Th17 responses and increased recruitment of granulocytes into the neuroretina. The STAT3 pathway modulates interactions between B and T cells during EAU, resulting in alteration of lymphocyte repertoires by increasing levels of autoreactive T cells while suppressing development and/or expansion of immunosuppressive lymphocytes (Bregs and Tregs) (48). At the genetic level, it has been reported that SLAMF5 is a negative modulator for IL-10^+^ Bregs and that blocking SLAMF5 could ameliorate EAE disease activity (49).

It should be noted that IL-10–producing Bregs made up only 1%–2% of the total B220^+^ splenic cells in mice and were not normally detectable in blood or lymph nodes (50). This low frequency is therefore a limitation for isolating and characterizing distinct Breg subsets in EAU. Our study identified that the B10 subset was detectable within the retina only after MTB priming or EAU induction. In contrast, T2-MZP and MZB cells were present solely in the spleen and T2-MZP cell levels positively correlated with EAU disease severity. Although our study focuses on Breg subsets at the peak of EAU, further research is needed to elucidate their dynamic roles in chronic and recurrent forms of the disease. The strength of our study lies in demonstrating that both the phenotypically distinct, B10-Breg subset in EAU and the iBreg subset in NIU suppressed T-cell proliferation and correlated with increases in expression of IL-10. Effective treatments for NIU are likely to require the modulation of complex interactions between Bregs, B10 cells, Teff cells and Tregs. Therefore, further investigation is needed before developing new B cell–directed therapies.

We also examined the relationship between gut microbiota, inflammation, and Breg subsets. It is known that short-term oral antibiotics modulate the severity of EAU by increasing Tregs in the gut and extraintestinal tissues, as well as ameliorating Teff cells and cytokines (34, 51, 52) and elimination of gut microbiota promotes the differentiation of IL-10–producing Bregs in the spleen and the mesenteric lymph nodes (44). In our study, the EAU + ABX mice developed milder inflammation compared with the EAU mice without ABX pre-treatment (Fig. 2a–c), highlighting the effect of commensal gut microbiota on EAU development. In autoimmune disease, microbiota regulation of host immunity plays an important role in B-cell activation. It has been reported for MS that helminth infection led to an increased frequency of IL-10^+^ B1 cells as well as a neuroprotective effect. This result may be due to IL-10–producing B1 cells, induced by helminth infection, suppressing T-cell proliferation and IFNγ production (53). In our study, gut microbiota depletion selectively promoted splenic IL-10–producing B10 Bregs, but not IL-10–producing splenic B1, T2-MZP, and MZB cells in EAU, suggesting the splenic IL-10–producing B10 Bregs may have a specific role in regulating disease activity. Levels of IL-10–producing B10-Bregs in PB were very low and decreased further in response to ABX, suggesting that gut microbiota are driving splenic IL-10–expressing Bregs as part of natural immunity. Salvador *et al*. demonstrated that administering ABX long term prior to EAU induction alters intestinal intraepithelial lymphocytes, disrupting a regulatory mechanism that normally offsets the EAU-ameliorating effects of microbiota depletion. Future research is required to investigate how specific microbial components mediate this regulatory effect on EAU and their association with regulatory B-cell subsets (52).

In the MTB-immunized control mice, we detected increases in IL-10–expressing B10-Breg subsets in blood, but not in the spleen or retina tissue. This was an unexpected finding since these mice do not exhibit any overt inflammatory response and highlights the importance of including sham controls. The fact that splenic EAU + ABX B10-Bregs were similar to the EAU + NAB B10-Bregs in their capacity for downregulating Teff-cell responses was unexpected. This might be due to the fact that when comparing equal numbers of Bregs, the B10 Bregs demonstrated a similar immunosuppressive capacity (Fig. 5) but that there were increased proportions of IL-10–expressing B10 Bregs in EAU + ABX mice (Fig. 3c) and offers an explanation as to why EAU was clinically less severe following ABX treatment. It suggests the *in vivo* regulatory capacity of B10-Bregs was microbiome independent but the increased frequency of B10 Bregs was microbiome dependent. The hypothesis is that, when comparing the B10 Bregs from the different groups at the single cell level, B10-Bregs from both EAU groups were more effective inhibitors due to their spleens being enriched for antigen-stimulated Bregs *in vivo* which might be more immunosuppressive.

TGF-β is another cytokine produced by Bregs that is detectable within the eye (48). The phenotypes of TGF-β^+^ Bregs are poorly characterized, but they are thought to induce anergy in T cells (54). IL-35 is an anti-inflammatory cytokine from the IL-12 family which is another Breg-secreted immunoregulatory cytokine involved in interstitial lung disease related to systemic sclerosis (54,55). IL-35–producing Bregs were identified in EAU as having an anti-inflammatory role (56). Unfortunately, we did not include TGF-β or IL-35 in our study since the focus was to address which subsets of Bregs were detectable in retinal inflammatory disease and their ability to inhibit CD4^+^ T cell responses. However, the immunoregulatory cytokines produced by Breg subsets are likely to be dictated by various factors, including the disease target tissue, the cells’ microenvironment and the genetic background of the host, resulting in some Breg populations preferentially secreting IL-10 or IL-35. Interestingly, although the markers of Bregs are not exactly the same for human cells as those used for mice, the iBreg subset in man was identified as a precursor of B10 cells and served as a biomarker for disease resolution. Similarly, in EAU, only B10 Bregs were decreased at peak disease and could be used as an indicator for EAU disease activity. Future studies should investigate the potential role(s) for Breg subsets during disease relapses using models of recurring disease. An additional limitation includes that the individual effects of immunization components, including antigens, adjuvants, and innate immune stimulators, on Breg subset induction and function were not examined. Defining how these factors shape Breg responses may provide further mechanistic insight into retinal immune regulation.

In conclusion, although present at relatively low levels, functionally active iBregs and B10 Bregs are likely to be involved in disease resolution in NIU and in EAU, respectively. Understanding the role of Breg subsets involved in NIU could improve clinical management if these cells were targeted therapeutically for personalized medicine.

## Supplementary Material

dxag021_Supplementary_Data
