# Supplementary material for: Selective decreases in regulatory B-cell (Breg) subsets in active uveitis: IL-10+CD19+CD24hiCD38hiBregs in patients and B10 Bregs in mice
Source: Int Immunol. 2026 May 7;38(9):569–80. doi: 10.1093/intimm/dxag021 (PMC13529577; doi:10.1093/intimm/dxag021)
Supplement: Supplementary file 1 [file dxag021_supplementary_data.zip › Supplementary figure caption.docx]

**Supplementary Figure 1.** Representative flow cytometry gating strategy to identify live (A), single cells (B), lymphocytes (C), CD19- expressing B cells (D), CD24 and CD38 expression (E, F) and CD19^+^CD24^hi^CD38^hi^ immature regulatory B cell (iBreg), CD19^+^CD24^hi^CD38^lo^-primary memory B cells, and CD19^+^CD24^int^CD38^int^-primary mature B cells in patients’ blood (G); IL-10-expression by isolated iBreg cells at 2, 6 months’ post treatment (H).

**Supplementary Figure 2.** Representative flow cytometry gating strategy of CD19^+^ B cells to identify B10-CD19^+^CD1d^hi^CD5^+^ and B1-CD19^+^CD5^+^ cells. (A) Fluorescence Minus One (FMO) of CD1d control. (B) FMO CD5 control. (C) Representative results of B10 cells in EAU+NAB group. (D) Representative results of B10 cells in NI group. (E) Scatter plot demonstrating the IL-10-expressing CD19^+^ B cells in intraocular single cells from an EAU ABX group mouse. (F) IL-10 expression in B10 Bregs in EAU+NAB and EAU+ABX groups. Representative data of three independent experiments are shown. Data were presented as mean ± SEM. **P* < 0.05 using a two-tailed, unpaired Student’s t test.

**Supplementary Figure 3**. Representative sorting strategy of CD19^+^CD24^hi^CD38^hi^ Breg cells in human peripheral blood study.

**Supplementary Figure 4.** Sorting and gating strategy of EAU CD19^+^CD1d^hi^CD5^+^ regulatory B10 cells and T effector cells (CD3^+^CD4^+^CD25^-^CD127^lo^) by flow cytometry.

**Supplementary Figure 5.** Preliminary data demonstrating the specific microbiome species depletion before (A) and after (B) broad spectrum antibiotics treatment in B10.RIII EAU mice. *Lactobacillus species* are a major part of the normal gut microbiota in mammals. Prior to antibiotics treatment, the total bacteria load was 1.87 × 10^9^ colony per gram (cpg) of feces with *Lactobacilli spp* being the majority. After 7-days’ antibiotics, the total bacteria colonies reduced to less than 300 cpg of faeces. Colonies of *Lactobacillus spp., Streptococcus intermedius,* and *Staphylococcus xylosus* decreased to less than 100 colonies per gram of faeces. Representative data of three independent experiments are shown. n=5 in each treatment group.
